# The first study of a mentalizing mediator model in a community sample of nonsuicidal self-injured adolescents and its association with salivary oxytocin

**DOI:** 10.3389/fpsyt.2025.1590441

**Published:** 2025-08-29

**Authors:** Chengjing Chu, Yanhua Zhou, Zhixiong Lin, Zihan Wang, Jie Zhang, Yibin Lu

**Affiliations:** ^1^ Department of Psychology, School of Humanities and Management, Guangdong Medical University, Dongguan, China; ^2^ Affiliated Brain Hospital of Guangzhou Medical University, Guangzhou, China; ^3^ The Affiliated Hospital of Guangdong Medical University, Zhanjiang, China; ^4^ Department of Child & Adolescence Psychology, Zhongshan Third People’s Hospital, Zhongzhan, China; ^5^ Department of Child & Adolescence Psychology, Dongguan Seventh People’s Hospital, Dongguan, China

**Keywords:** mentalizing, childhood trauma, salivary oxytocin, nonsuicidal self-injury, structural equation modeling

## Abstract

**Background:**

Nonsuicidal self-injurious behavior (NSSI) is highly prevalent in adolescents and strongly associated with early trauma. Emerging theories have indicated that the occurrence of NSSI results from the interaction of individual biological vulnerability and environmental risk; however, the underlying mechanisms are not yet clear. This study sought to investigate the psychological and pathophysiological mechanisms underlying the development of self-injury behavior through environmental, psychological, and physiological factors. We hypothesized that mentalization mediates the relationship between childhood trauma and NSSI, and further explored whether oxytocin (OT) has the potential to serve as an informative biomarker of social functioning for people with NSSI.

**Methods:**

This study investigated NSSI, childhood trauma, and mentalizing in 1313 junior high school students to develop and test a mediated model of mentalizing in which childhood trauma affects NSSI. Subsequently, the relationship between peripheral salivary OT levels and NSSI in a cohort of 109 individuals with suicidal self-injurious behavior and 113 healthy controls.

**Results:**

The NSSI detection rate was 28.2% in this study. Females had a greater frequency of NSSI, hypomentalization and lower hypermentalization.The structural equation modeling (SEM) results revealed that the indirect effect of childhood trauma on NSSI through hypomentalization was 0.091 (95% CI [0.066, 0.120], P< 0.001). The indirect effect of childhood trauma on NSSI through hypermentalization was 0.037 (95% CI [0.025, 0.049], P < 0.001). OT levels were not significantly correlated with hypermentalization, hypomentalization, or childhood trauma (*P* > 0.05).

**Conclusions:**

The present study revealed that mentalizing partially mediated the associations between childhood trauma and NSSI, suggesting that the mentalizing trauma model is equally applicable to the community NSSI population. This is the first study to explore the relationship between peripheral OT levels and NSSI behavior, with results suggesting that baseline salivary OT concentrations are not reliable biomarkers for NSSI in community samples.

## Introduction

Nonsuicidal self-injurious (NSSI) behavior is closely associated with psychiatric disorders, such as personality disorders, particularly borderline personality disorder (1), and self-harm is a major symptom of Borderline Personality Disorder (BPD) (2). NSSI is also an important predictor of suicide, and previous studies have often examined the mechanisms of suicide and NSSI together (3). However, the Diagnostic and Statistical Manual of Mental Disorders, 5th edition (DSM-5), independently identifies NSSI as a diagnostic category for psychiatric disorders, and although the two are closely related (4), some studies have confirmed differences in their pathogenesis (5, 6). The prevalence of NSSI among adolescents has been increasing rapidly in recent years, and research on its mechanisms has received increasing amounts of attention.

The concept of mentalizing was first introduced in people with BPD. Mentalizing reflects an individual’s ability to visualize abstract cues to perceive abstract cues, such as tone of voice and facial expressions, in oneself and others, and to process one’s own cognitive ability to learn about both one’s own and others’ mental states (including intentions, desires, emotions, and beliefs) and infer the behavior of others (7). Mentalizing is considered to be a more complex and advanced cognitive function (8). Fonagy proposed that reflective functioning (RF) could be used to measure mentalizing ability and reported that impaired RF was associated with borderline symptoms in adolescents. He also proposed a model of mentalizing development (9), suggesting that BPD increases the risk of impaired mentalizing when experiencing early trauma (10). Patients with BPD frequently confuse reality with subjective imagination when perceiving the inner and outer worlds, struggle to set boundaries between external reality and the inner world in understanding the social world, manifest as self-centered, experience fragmentation in their interactions with others, and undergo identity-self dissociation. These challenges increase the risk of undesirable emotions and behaviors, such as self-harm, in an attempt to change their current social relationships.

Childhood is an essential period for the development of mentalizing skills. Trauma during childhood, which exceeds the capacity for psychological regulation, can severely affect the development of mentalizing abilities during adolescence, leading to disruptions in mentalizing abilities and distortion of one’s cognitive state and the understanding of others in social interactions, causing interpersonal distress and increasing the risk of self-injury (11, 12). Neurodevelopmentally, childhood trauma acts a negative stressor that increases susceptibility to NSSI, as it disrupts the structure and function of the corresponding brain regions. The use of maladaptive behaviors (e.g., NSSI) is a maladaptive strategy used by adolescents who have experienced childhood trauma to cope with environmental interpersonal stress. Individuals with early adverse experiences are inclined to overanalyze the mental states of others, and such negative interpersonal interactions can activate the individual’s attachment system increasing emotional arousal to a level that temporarily inhibits mentalizing and may lead to acting out through self-harm (13). In a nonclinical sample, individuals with high externalizing symptoms (e.g., violence, impulsivity) were found to have lower RF scores (14), implying that mentalizing deficits were significantly associated with psychopathic traits as well as active aggressive behavior (15). The present study hypothesized that impaired mentalizing may be a potential risk for the occurrence of ambulatory NSSI in individuals exposed to childhood trauma, providing new insights into the mechanisms of NSSI.

Previous pro-social effects of oxytocin (OT) and its ability to promote empathy have been extensively reported in animal and human behavior studies. Studies have shown that OT deficiency, abnormal metabolism, and dysfunctional OT receptors are significantly associated with self-injurious behavior, social interpersonal problems, emotional dysregulation, and depression (16, 17). Ditzen et al. (18) reported that OT was related to pro-social behavior in adolescents. Pro-social behavior increases adolescents’ willingness to cooperate with and trust others, enhances their willingness to communicate, and promotes interpersonal skills, thus helping to reduce the occurrence of NSSI behaviors (18). OT is a social cognitive physiological hormone that improves social cognition and fosters positive social relationships (19). Recent studies have revealed reduced OT in BPD patients, who are at greater risk for self-harm and mentalizing deficits (20–22).

This study hypothesized that mentalization and OT influenced the risk of NSSI at the psychosocial and physiological levels, respectively. Therefore, this study investigated the pathogenesis of NSSI in adolescents through a combination of sociophysiological, psychological, and environmental factors in an ambulatory population, providing a scientific basis and theoretical foundation for the detection and prevention of NSSI.

The first part of this two-part study examines whether mentalizing deficits are involved in the pathogenesis of NSSI from childhood trauma through a mediation model. The second part explores whether the biological factor OT is involved in the pathological process of NSSI through a case-control study.

## Materials and methods

### Participants

The participants were recruited from three middle schools in Dongguan, Guangdong Province, China, with the participating students aged 11–15 years. The participants were guided by pretrained data collectors to complete paper questionnaires in the classroom. The Adolescent Nonsuicidal Self-injury Assessment Questionnaire (ANSAQ) was used to complete the basic screening for NSSI situations. A total of 1400 questionnaires were distributed, and 1313 valid questionnaires were returned, yielding an effective recovery rate of 93.93%. Among the participants, 668 were male students, 645 were female students, 938 were in the first year of junior high school, and 375 were in the second year of junior high school. A total of 109 NSSI individuals and 113 healthy controls were recruited from these screening samples, and saliva was collected at a uniform date and time point to complete the case-control study. In the NSSI group (109 participants), 53 participants were male and 56 were female. In the non-NSSI group (113 participants), 55 participants were male and 58 were female. The study was authorized by the Research Ethics Committee of Guangdong Medical University. All participants signed an informed consent form, and consent was obtained from guardians and class teachers. The ethical approval number is PJKT2022-051.

### Scale assessment tools

#### Adolescent nonsuicidal self-injury assessment questionnaire

The Chinese version of the NSSI behavior questionnaire for adolescents, developed by Liu et al. (23), was selected to obtain the occurrence of NSSI among the participants in the past year. This scale consists of 2 subquestionnaires: the behavioral questionnaire and the functional questionnaire. The behavioral questionnaire included 12 forms of injury, including intentionally yanking one’s own hair, intentionally biting, and cutting oneself, among others. The number of occurrences of self-injurious behaviors was used as the overall self-injury frequency. The higher the total self-injury score, the more severe the self-injury. In accordance with the Diagnostic and Statistical Manual of Mental Disorders, 5th edition (DSM-5) criteria, subjects with a frequency > 5 times were judged as the “self-injury group.” The functional questionnaire contains 19 items, each with a five-point scale ranging from 0 (not at all) to 4 (fully). In this study, positive entries were defined as those resulting from 3 (consistent) and 4 (fully consistent). One or several positive entries in each dimension were defined as having an NSSI function. In this study, simultaneous self-injury frequency >5 times and at least one positive NSSI function were classified as NSSI enrollment criteria. The Cronbach’s α values of the behavioral and functional questionnaires in this study were 0.862 and 0.885, indicating good internal consistency, respectively.

#### Assessment of mentalization level

The simplified Chinese version of the Reflective Functioning Questionnaire-8 (RFQ-8) by Xu (24) was selected to assess the level of mentalization of secondary school students.The same four items were used to calculate the scores for both subscales, with these items being reverse-scored between RFQ_C and RFQ_U (e.g., “I don’t always know why I do what I do”; “When I’m angry, I say things I later regret”; “If I’m unsure of myself, I may act in ways that offend others”; “Sometimes I do things without exactly knowing why”). The remaining four items (two for each subscale) were unique to their respective subscales (e.g., for RFQ_C: “When I’m angry, I say harsh words without knowing why I said them”; for RFQ_U: “I always know what I’m feeling”). The scale consists of eight items, with responses ranging from “strongly disagree” to “strongly agree,” scored as “0,0,0,0,0,1,2,3” and “3,2,1,0,0,0,0.”. Hypermentalization reflects high levels of imagination and reasoning, while hypomentalization is the opposite, lacking objective content and an inability to understand one’s own and others’ mental states. Higher scores on the certainty subsection scale and lower scores on the uncertainty subsection scale indicate greater mentalizing ability. Conversely, lower scores on the certainty subsection scale and higher scores on the uncertainty subsection scale indicate greater deficits in mentalizing ability. The Cronbach’s α for RFQU was 0.691, and for RFQC, it was 0.793, indicating adequate reliability.

#### Childhood trauma questionnaire

The CTQ was constructed by Bernstein et al. (25), a clinical psychologist in New York, USA, as an instrument to assess traumatic experiences in childhood. It contains 28 items, with each subscale containing five items, and each item is rated on a 5-point scale (1, 2, 3, 4, 5 for never, occasionally, sometimes, often, always, respectively), with each abuse subscale scoring 5–25 and a total score of 25–125, with higher scores indicating higher levels of trauma. The subscales were composed of emotional abuse (EA), physical abuse (PA), sexual abuse (SA), emotional neglect (EN) and physical neglect (PN). In this study, Cronbach’s α was 0.605.

### OT collection and detection

#### Saliva collection

Each participant was instructed not to eat or drink anything for 2 hours prior to saliva collection. Prior to the measurement, each participant was permitted to rest quietly in a room (outside the test room) for 30 min. They were then taken to the nearby room, where a baseline saliva sample was collected with a cotton roll (Neutral Salivettes^®^, SARSTEDT, Numbrecht, Germany), while being monitored by a laboratory technician. The participants held the cotton roll in their mouths, performing circular movements to avoid soaking the cotton, prior to replacing it with the stopper part of the Salivette^®^ tube. Saliva samples were stored at −20°C until they were centrifuged twice, 2 days apart, at 40°C at 1,500 × g for 20 min. Liquid samples were stored at −80°C.

#### OT detection

On the day of the assay, the samples were redissolved in liquid and concentrated four times in buffer before immunoassay with a Human Oxytocin ELISA Kit (catalog #MK0807A; Sumeike Biological Technology Co., Jiangsu, China). Measurements were performed in duplicate, and the concentration was calculated via a microplate reader (RT-6100, China) according to relevant standard curves.

### Quality control

The collectors were trained uniformly via uniform guidelines and were informed of the principle of confidentiality. The questionnaires and saliva samples were collected in a standardized manner via collective administration. Mediated effects tests were performed with the bootstrap method, with random sampling of the original sample 5000 times to redistribute the samples and obtain robust standard errors and confidence intervals for the parameter estimates. A 95% CI not containing 0 was considered a statistically significant difference. The test level was set at a=0.05.

### Data analysis

Preliminary analysis revealed that the OT and questionnaire data were not normally distributed. The nonnormally distributed measures were expressed as M (P25, P75), and the two groups were compared via the Mann–Whitney U test, depending on the type of variable. Spearman’s rank correlation coefficient was used to assess associations between OT levels, sociodemographic variables, and questionnaire data. Mediated effects analysis and model fitting were performed via AMOS 24.0. Model fit was assessed based on the following criteria: the degrees of freedom (χ2/DF) < 3, the goodness-of-fit index (GFI) > 0.90, the adjusted goodness-of-fit index (AGFI) > 0.90, the comparative fit index (CFI) > 0.90, the incremental fit index (IFI) > 0.90, the Tucker–Lewis coefficient (TFI) > 0.90, the standardized root mean square residual (SRMR) <0.08, and the root mean square error of approximation (RMSEA) < 0.08. If the model met these criteria, it was considered to fit well.

## Results

### Common method deviation test

The Harman one-factor test was used to control the procedure (e.g., reverse scoring of some items). All the collected question items were tested for common method bias via Harman’s one-way test, and the results of the unrotated exploratory factor analysis extracted a total of 14 factors with characteristic roots greater than 1. The maximum factor variance explained was 19.76% (< 40%) for this study, so there was no serious common method bias in this study.

### Detected situation and frequency of nonsuicidal self-injury

Among the adolescents surveyed, the self-injurious behavior detection rate was 47.6% (625/1313), with 370 cases of self-injurious behavior occurring five times or more, and the NSSI detection rate was 28.2% (**Table 1**).

**Table 1:** Distribution of the frequency of NSSI behaviors.

Frequency of self-injury	Number of cases (n)	Composition ratio (%)
0 time	688	52.4
1 time	92	7
2 times	71	5.4
3 times	64	4.9
4 times	28	2.1
≥5 times (NSSI)	370	28.2
<5 times (Non-NSSI)	943	71.8

NSSI, Nonsuicidal self-injurious.

### Mann–Whitney U tests for sex and grade level in this study

There were significant sex differences (P < 0.01, **Table 2**) in hypermentalization, hypomentalization, and NSSI behavior. Females had a greater frequency of NSSI, hypomentalization and lower hypermentalization; the grade level differences were not significant (P > 0.05).

**Table 2:** Tests for differences in grade and gender.

Groups	Childhood trauma	Hypermentalization	Hypomentalization	NSSI frequency
Male (n=668)	44 (37–52)	4 (1–10)	5 (2–8.5)	2 (0–11.75)
Female (n=645)	44 (36.75–52)	3 (1–7)	4 (2–8)	0 (0–12.25)
Z	−1.199	−5.604	−2.265	−3.692
*P*	0.230	< 0.001	0.023	< 0.001
Grade 7 (n=938)	41 (37–48)	4 (2–7)	0 (0–5)	5 (2–9)
Grade 8 (n=375)	42 (36–48)	4 (2–7)	0 (0–5)	5 (1–9)
Z	−0.449	−0.391	−0.277	−0.712
*P*	0.653	0.696	0.782	0.477

NSSI, Nonsuicidal self-injurious; Z, Mann–Whitney U test; same as the following.

### Correlation analysis of childhood trauma, mentalizing, and frequency of nonsuicidal self-injurious behavior

The Spearman rank correlation test was used due to the non-normal distribution of the measurements. The results (**Table 3**) revealed that hypomentalization was positively correlated with total childhood trauma scores, scores for various trauma type, and NSSI behaviors at significant levels (P < 0.001). Hypermentalization was negatively associated with childhood trauma, scores for various trauma types (except SA), and NSSI behavior at significant levels (P < 0.001). Childhood trauma and various trauma types were positively associated with NSSI (P < 0.001).

**Table 3:** Correlation analysis of childhood trauma, mentalizing, and NSSI frequency.

Variable	1	2	3	4	5	6	7	8	9
1. NSSI	1								
2. RFQU	0.416***	1							
3. RFQC	−0.271***	−0.557***	1						
4. EN	0.287***	0.241***	−0.177***	1					
5. PA	0.341***	0.239***	−0.145***	0.344***	1				
6. EA	0.504***	0.343***	−0.239***	0.436***	0.590***	1			
7. SA	0.123***	0.092***	−0.037	0.093***	0.321***	0.306***	1		
8. PN	0.266***	0.199***	−0.161***	0.622***	0.338***	0.385***	0.160***	1	
9. CTQ	0.438***	0.330***	−0.233***	0.778***	0.700***	0.764***	0.377***	0.727***	1

NSSI, Nonsuicidal self-injurious; CTQ, childhood trauma; RFQU, Hypomentalization; RFQC, Hypermentalization; EA, emotional abuse; PA, physical abuse; SA, sexual abuse; EN, emotional neglect; PN, physical neglect; ***P< 0.001.

### Regression analysis of mentalizing and NSSI frequency in adolescent childhood trauma

In this study, multiple linear regression was performed to further explore the relationships among childhood trauma, mentalizing, and NSSI among junior high school students. The total childhood trauma score, each trauma dimension, and each dimension of mentalizing were used as independent variables, with NSSI behaviors as the dependent variables.

Regression analysis revealed that hypermentalization (β = −0.062, t = −2.142, P < 0.05) significantly and negatively predicted NSSI behavior. PA (β = 0.061, t = 2.055, P < 0.05), EA (β = 0.446, t = 14.465, P < 0.01), childhood trauma (β = 0.440, t = 17.742, P < 0.01) and hypomentalization (β = 0.382, t = 12.651, P < 0.01) were significant positive predictors of NSSI behavior. The EN, SA, and PN dimensions of childhood trauma did not significantly affect NSSI behavior (P > 0.05) (**Table 4**). Among them, the SA dimension of childhood trauma did not significantly impact NSSI behavior, which may be related to the low incidence of sexual abuse, resulting in insufficient statistical power.

**Table 4:** Regression analysis of early trauma and mentalizing on the frequency of nonsuicidal self-injury.

Dependent variables	Predicted variables	*β*	*t*	*P*	*R*	*R_j_^2^*	*F*
NSSI	EN	0.042	1.342	0.180	0.518	0.266	96.006^***^
	PA	0.061	2.055	0.04			
	EA	0.446	14.465	0.001			
	SA	−0.045	−1.759	0.079			
	PN	0.056	1.805	0.071			
NSSI	CTQ	0.440	17.742	0.001	0.440	0.193	314.764^***^
	RFQU	0.382	12.651	0.001			
NSSI	RFQC	−0.062	−2.142	0.032	0.422	0.177	141.905^***^

The table contains three different models. NSSI, Nonsuicidal self-injurious; CTQ, childhood trauma; RFQU, Hypomentalization; RFQC, Hypermentalization; EA, emotional abuse; PA, physical abuse; SA, sexual abuse; EN, emotional neglect; PN, physical neglect; ***P < 0.001.

### Analysis of the intermediary model

#### SPSS process macro procedure bootstrap method test

To explore this further, this study conducted a mediated effects test using childhood trauma and its dimensions as independent variables, NSSI frequency as the dependent variable, and two dimensions of mentalizing as mediating variables, controlling for gender. The nonparametric percentile bootstrap method, advocated by Zhonglin Wen (26), was used. A bootstrap sample of 5000 was first drawn, with robust standard parameter errors and confidence intervals estimated, using a 95% CI excluding 0. The sample was tested for mediation effects via the SPSS Process macro program prepared by Hayes.

The bootstrap method-mediated effects test revealed that adolescents’ hypermentalization had significant mediating effects on EN, PA, EA, PN, CTQ, and NSSI, with the ratios of indirect effects to total effects being 14.04%, 9.66%, 7.6%, 8.18%, 14.18%, and 9.50%, respectively. The mediating effect between adolescent hypomentalization and CTQ was significant for all dimensions and NSSI, with mediating effect sizes ranging from 18.73% to 31.03%; none of their confidence intervals were 0.03%. In addition, the mediating effect of hypermentalization between SA and NSSI was not significant, with bootstrap intervals including 0 (**Table 5**).

**Table 5:** Test of the mediating effect of mentalizing between childhood trauma and NSSI.

Independent variable	Mediating variables	Indirect effect value	SE	95% CI		Intermediary effect ratio
				Lower	Upper	
EN	RFQC	0.0645	0.0112	0.0432	0.087	14.04%
PA		0.1119	0.0208	0.0719	0.1533	9.66%
EA		0.0958	0.0139	0.0696	0.1244	7.60%
SA		0.0747	0.0551	−0.0271	0.1914	8.18%
PN		0.1118	0.0197	0.0746	0.1528	14.18%
CTQ		0.0335	0.0048	0.246	0.0434	9.50%
EN	RFQU	0.1425	0.0227	0.0997	0.19	31.03%
PA		0.2877	0.0505	0.1967	0.3929	24.83%
EA		0.236	0.0347	0.172	0.3095	18.73%
SA		0.2783	0.1086	0.0957	0.5243	30.47%
PN		0.2234	0.0401	0.1501	0.3068	28.34%
CTQ		0.0805	0.0119	0.0588	0.1053	22.91%

SE, standard error; NSSI, nonsuicidal self-injurious; CTQ, childhood trauma; RFQU, hypomentalization; RFQC, hypermentalization; EA, emotional abuse; PA, physical abuse; SA, sexual abuse; EN, emotional neglect; PN, physical neglect.

### Structural equation model testing

Using structural equation modeling (SEM) tests can make the results of bootstrap tests a more convincing and scientific exercise. To test the mediating model that exists between childhood trauma, mentalizing, and NSSI, we used SEM.

Hypomentalization and hypermentalization are inversely correlated with childhood trauma and NSSI, respectively. In this study, the two dimensions of mentalizing are modeled separately as mediating variables, and the hypotheses that they mediate the relationship between childhood trauma and NSSI were tested individually.

**Figure 1:**
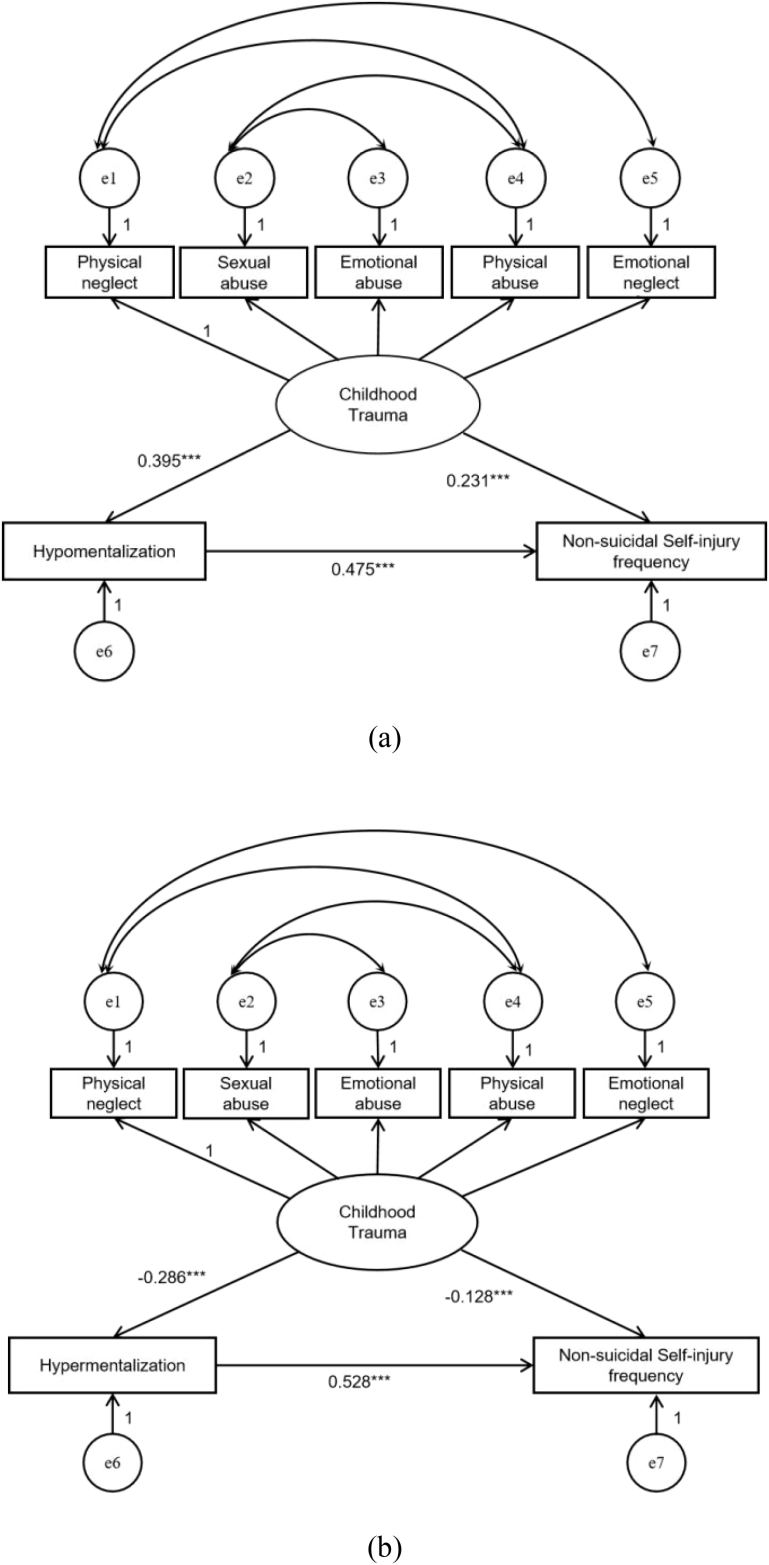
Pathways related to childhood trauma, hypomentalization, and NSSI frequency. **(a)** Modified model 1. **(b)** Modified model 2. (Note: ***P < 0.001).

Model 1: Structural equation modeling was used to verify the mediating role of mentalizing deficits in the association between childhood trauma and NSSI. The model fit well after model correction. All of the childhood trauma → hypomentalization → NSSI pathways were statistically significant. Childhood trauma positively predicted hypomentalization (β = 0.395, P < 0.001), hypomentalization positively predicted NSSI frequency (β = 0.231, P < 0.001), and childhood trauma positively predicted NSSI frequency (β = 0.475, P < 0.001), as shown in **Figure 1a**. The results of the bootstrap method test indicated that the indirect effect of childhood traumatic experiences on NSSI was 0.091 (95% CI: 0.066–0.120), the total effect was 0.566 (95% CI: 0.501–0.631), the mediating effect accounted for 16.08% of the total effect, and hypomentalization was significantly and represents a partial mediating effect.

Model 2: Structural equation modeling was applied to verify the mediating role of hypermentalization between childhood trauma and NSSI. After model correction, when 3 < CMIN/Df value < 5, the model did not fit well (**Table 6**). The pathway of childhood trauma → hypermentalization → NSSI was statistically significant. Childhood trauma negatively predicted overmentalization (β = −0.286, P < 0.001), hypermentalization negatively predicted NSSI frequency (β = 0.528, P < 0.001), and hypermentalization could be used as a protective factor against NSSI. Childhood trauma positively predicted NSSI frequency (β = -0.128, P < 0.001) (**Figure 1b**). The bootstrap method test revealed that the indirect effect of childhood traumatic experience on NSSI was 0.037 (95% CI: 0.025–0.049), the total effect was 0.564 (95% CI: 0.499–0.629), and the mediating effect accounted for 6.56% of the total effect. The mediating effect was significant and partially mediated, represents a partial mediating effect. However, the model fit was average (3 < CMIN/DF < 5).

**Table 6:** Model fit superiority index.

Fitting index	*χ* ^2^	*P*	CMIN/DF	GFI	AGFI	SRMR	CFI	TFI	RMSEA
Original model 1	418.242	0.001	32.172	0.917	0.822	0.070	0.830	0.725	0.154
Original model 2	388.308	0.001	29.870	0.919	0.825	0.067	0.814	0.700	0.148
Modified model 1	25.562	0.002	2.840	0.995	0.983	0.018	0.993	0.984	0.037
Modified model 2	29.229	0.001	3.248	0.994	0.981	0.211	0.991	0.979	0.041

The above analysis verified that childhood trauma can influence NSSI behavior through mentalizing, and although the model of hypermentalization exhibited a fair fit, the model of hypomentalization demonstrated a good fit. Furthermore, hypermentalization can influence NSSI risk partially through childhood trauma.

### Comparison of OT concentration and mentalizing ability between the NSSI and non-NSSI groups and correlation analysis

The differences in OT concentration between the NSSI and non-NSSI groups were not significant, and the differences in hypermentalization, hypomentalization, and childhood trauma scores between the two groups were statistically significant (P > 0.05, **Table 7**). Correlation analysis revealed that OT concentration was not significantly correlated with childhood trauma, NSSI frequency, hypermentalization, or hypomentalization (P > 0.05, **Table 8**).

**Table 7:** Comparison between the two groups.

Variables	NSSI (n = 109)	Non-NSSI (n = 113)	Z	*P*
Oxytocin	130.56 (102.2–158.615)	132.4 (110.11–155.475)	−0.222	0.825
Hypomentalization	7 (4–10)	3 (1–5)	−6.596	< 0.001
Hypermentalization	3 (0–5)	5 (2–12)	−4.518	< 0.001
Childhood trauma	49 (41–60)	39 (35–45)	−7.27	< 0.001
Emotional neglect	13 (9–17)	9 (5.5–12)	−5.29	< 0.001
Physical abuse	7 (5–9)	5 (5–6)	−5.311	< 0.001
Emotional abuse	9 (6.5–13)	5 (5–7)	−7.197	< 0.001
Sexual abuse	5 (5–5)	5 (5–5)	−2.606	0.009
Physical neglect	9 (6–11)	6 (5–8)	−5.048	< 0.001

**Table 8:** Correlation analysis with OT.

Vorables	1	2	3	4	5
1. Oxytocin	1				
2. NSSI frequency	−0.007	1			
3. Hypermentalization	−0.022	−0.345^**^	1		
4. Hypomentalization	−0.011	0.497^**^	−0.663^**^	1	
5. Childhood trauma	−0.024	0.530^**^	−0.311^**^	0.444^**^	1

NSSI, Nonsuicidal self-injurious, **P < 0.01.

### Predicted NSSI risk via binary logistic regression analysis

In terms of age and sex, binary logistic regression analysis revealed that the CTQ score positively predicted NSSI risk (OR = 1.094, 95% CI: 1.054–1.135, P < 0.001), the RFQU score positively predicted NSSI risk (OR = 1.182, 95% CI: 1.060–1.318, P = 0.003), and the OT concentration had no significant effect on NSSI risk (P < 0.05) (**Table 9**).

**Table 9:** The impact of childhood trauma, mentalizing, and OT on NSSI.

Vorables	B	SE	wals	*P*	OR	95% CI	
						Lower	Upper
CTQ	0.090	0.019	22.486	<0.001	1.094	1.054	1.135
RFQU	0.167	0.056	9.070	0.003	1.182	1.060	1.318
RFQC	−0.022	0.042	0.2709	0.604	0.978	0.901	1.063
Oxytocin	0.001	0.004	0.001	0.971	1.000	0.991	1.009
Sex	0.157	0.331	0.226	0.635	1.170	0.612	2.238
Age	−0.430	0.242	3.161	0.075	0.651	0.405	1.045

OR, odds ratio; CI, confidence interval; CTQ, childhood trauma; RFQU, hypomentalization; RFQC, hypermentalization.

## Discussion

Since COVID-19 and its countermeasures in worldwide have caused widespread mental health problems. Lots of data shew that psychological, economic, behavioral, and psychosocial problems associated with the COVID-19 pandemic may lead to a growth in self-harm (27, 28). In the screening sample, this study revealed an elevated rate of NSSI detection compared with that before the onset of COVID-19, which aligned with the results of a longitudinal study of NSSI in an Italian adolescent sample (29). These findings indicate that NSSI is prevalent in the adolescent population and deserves the attention of schools and professionals. However, the detection rate of NSSI in adolescents was higher by Xu et al. (30), which may be different from the original source of our sample. This study focused primarily on secondary schools in the city of Dongguan. The investigation revealed that the occurrence of NSSI nationally was influenced by urban culture (31).

As a rapidly emerging first-tier city, Dongguan has attracted a large number of non-local workers. Pressure from the economic downturn caused by the pandemic has had a significant impact on the families of migrant workers, increasing economic pressures and raising parents’ expectations for their children’s academic performance while simultaneously disrupting parent-child communication. As a result, the prevalence of non-suicidal self-injury (NSSI) among the adolescent group in this study was relatively high. In addition, the samples for this study were collected 18 months after the pandemic, during a period when the country had entered a phase of regular control. Schools had shifted to online learning, the range of activities available to students was restricted, and peer interactions were limited. These challenges led to higher levels of negative emotions, such as depression and anxiety, among the adolescents in this study compared to those in previous studies, thereby increasing the risk of developing NSSI (32), particularly in those with preexisting vulnerabilities (e.g., a history of NSSI or high levels of internalization) (29).

We also found significant gender differences in childhood trauma, mentalizing, and NSSI behaviors in this study. Compared with boys, girls had higher levels of childhood trauma, hypomentalization, and NSSI behaviors, a result that aligned with previous views (13, 33, 34). A study that investigated the prevalence of NSSI among 16,853 secondary school students in three Chinese cities reported that female adolescents were at greater risk of NSSI than were male adolescents, whereas one study reported up to three times greater risk (35, 36). A recent longitudinal study of NSSI behavior in adolescents revealed that females not only had a greater risk of NSSI than males but also had a greater frequency of NSSI and a longer duration of NSSI (37). This might be related to the biological functioning of women, who are susceptible to negative emotions due to differences in the brain structure of emotions. Emotional attribution theory suggests that when faced with a negative event, men identify the cause from the objective environment, attribute unpleasant feelings to external sources, and are prone to externalizing negative emotions, whereas women tend to criticize themselves, attribute negative experiences to their own inadequacies, are prone to ruminative thinking, accumulate negative emotions, and are inclined to resort to inappropriate emotional regulation (NSSI) to vent (38, 39).

This was the first study to investigate the mediating role of mentalizing in the relationship between childhood maltreatment and NSSI in a nonclinical sample of teenagers, aiming to examine the underlying mechanisms of the effects of childhood maltreatment on NSSI. In this study, NSSI was found to be associated with mentalizing through a survey of 1313 junior high school students, and a pathway analysis was conducted. The results showed that mentalizing had a mediating effect on the occurrence of NSSI in the mediating pathway through which childhood trauma affected NSSI. Consistent with the findings of a recent study of a mediating model of mentalizing in clinical and community-based self-harm samples (40), attachment and mentalizing were found to play unique mediating roles between childhood trauma and psychopathology in this study of self-harm patients, which included clinical samples. We extended this model to a community NSSI sample and tested the mentalizing model of NSSI via the bootstrap method of the SPSS Process macro and structural equation modeling models, with community adolescent NSSI occurrence associated with mentalizing impairment. These studies reveal the mechanisms underlying the associations between childhood trauma and BPD and NSSI, where early adverse experiences may cause recent mental distress. In the face of negative events, early traumatic experiences are often revisited as intense emotional experiences, and people go on to avoid normal related psychological experiences and instead regulate emotional distress through maladaptive behaviors (41). NSSI is considered a maladaptive emotion regulation strategy that addresses negative emotions and stress in the short term but impairs interpersonal functioning in the long term, and it is not recognized as such (42). Mentalization deficits could lead to adolescents lacking socially beneficial qualities such as trust and empathy in interpersonal relationships and easily distort the true intentions of others, thereby increasing emotional distress and increasing the risk of NSSI, which is consistent with the cognitive–emotional model view (43). When we are unable to properly read through the expressions displayed by others during social interactions or even fail to read such cues to empathize with others’ internal experiences, leading to increased interpersonal distress, this often directly contributes to the failure of their self-emotional regulation strategies, prompting them to adopt NSSI to alleviate negative emotions. Individuals who suffer early trauma, particularly psychological trauma, may be more likely to lose this protective factor to maintain social relationships in the face of sudden stress, as confirmed by Germine et al. (44). Childhood trauma is not conducive to the effective development of mentalizing, and impaired mentalizing capacity is associated with many self-harm-related psychiatric disorders (45). Impaired mental health in the face of stress increases the risk of various types of psychiatric distress, and psychiatric distress and symptoms are positively associated with, for example, NSSI and depressed mood (46). Therefore, professionals need to focus on adolescent childhood trauma reduce the incidence of NSSI in adolescents. However, exploring the mechanisms of NSSI in terms of only psychological factors does not fully capture its underlying complexity, and this study also examines OT as a potential biological factor contributing to NSSI.

To explore the associations of social hormones such as OT with NSSI and mentalizing, this study included 109 NSSI and 113 non-NSSI individuals in the primary screening sample for OT testing. The results revealed that there was no significant difference in salivary OT levels between NSSI and non-NSSI individuals. In addition, OT levels did not correlate with measures of hypermindfulness, mentalizing deficits, or childhood trauma. Although OT is associated with mental illness, its potential association with NSSI remains underexplored. Bertsch reported an inverse relationship between blood OT levels and aggression in a study focusing on blood OT levels (47), and although the main focus was on personality disorders, the results suggested that OT could reduce aggressive behavior. A recent review demonstrated that OT could be an important mediator in the intergenerational transmission of early adversity in BPD (48). These findings indicate that OT may mediate the effects of childhood trauma on mental disorders. In contrast, our findings found no difference in salivary OT levels between the NSSI group and the control group. There may be multiple reasons for these inconsistent results. First, in clinical samples, OT levels in BPD patients have also been found to be related to the severity of the illness (49), and there may be no difference in OT concentrations between patients with milder psychiatric illnesses and healthy individuals. The sample selected for this study was a nonclinical sample, so the course of the disease was not as severe as that of the inpatients, and the damage to the OT system took some time to appear at the peripheral level. Second, the OT peaks might have shown a certain circadian rhythm of secretion, and the time selected did not show peak values (50). Second, this study selected saliva samples, whereas changes in the OT concentration in cerebrospinal fluid are much more sensitive and better reflect facial perception but are not related to peripheral OT (51, 52). Third, lower levels of OT were observed in patients hospitalized for suicide than in the normal group (53), a change that was not observed in patients with NSSI. Childhood trauma may not be linked to oxytocin (OT) in patients with borderline personality disorder (BPD). In addition, these findings suggest that the neurobiological mechanisms of suicide differ from those of NSSI and that the occurrence of NSSI may not involve changes in the OT system. In patients with borderline personality disorder (BPD), childhood trauma shows no association with oxytocin (OT) levels. This study produced results that align with these findings. However, patients with BPD are more likely to interact with experiences of childhood trauma, often exhibiting abnormal OT responses in social situations, thereby influencing changes in OT levels (54). Childhood trauma might not be sufficient to impair the function of OT but is also influenced by other factors, such as genes and personality traits, that jointly influence OT alterations.

Although OT cannot yet be considered as a candidate biomarker for NSSI, further evaluation in the NSSI population is needed in the future.

## Conclusion

This study developed a mentalizing model of NSSI in a community adolescent sample, extending the mentalizing model of BPD to community adolescents with NSSI and providing new ideas for mechanistic research and psychological interventions for NSSI. The case-control study revealed that baseline salivary OT concentrations did not serve as a biomarker for NSSI in community samples. This study was also the first to explore the association between OT concentrations and NSSI in a community-based sample of adolescents. Although salivary OT may not serve as a peripheral biomarker for NSSI, further evaluation is needed in adolescents with inpatient NSSI. Future studies can be exploratory and validated with a focus on the severity of NSSI.

## Limitation

The sample selected for this research consists of cross-sectional data, and future studies should include additional longitudinal data to enhance the credibility of the causal relationship between mentalizing and NSSI. With respect to the exploration of the biological factors of NSSI, although salivary OT is not associated with NSSI, the connection between central OT and NSSI cannot be eliminated, and future studies should investigate this further.

## Data Availability

The raw data are publicly available in the Figshare repository under doi: 10.6084/m9.figshare.29941013.
